# Single-cell transcriptome and surfaceome profiling of the adult human retinal pigment epithelium

**DOI:** 10.1016/j.stemcr.2025.102611

**Published:** 2025-08-28

**Authors:** Farhad Farjood, Swapna Nandakumar, Taylor Bertucci, Thomas Kiehl, Steve Lotz, Yue Wang, Jacob Black, Skanda Sai, Jade Kozak, Brigitte L. Arduini, Sally Temple, Nathan C. Boles, Jeffrey H. Stern

**Affiliations:** 1 Neural Stem Cell Institute, 150 New Scotland Ave., Albany, NY 12208, USA

**Keywords:** RPE, retinal pigment epithelium, AMD, age-related macular degeneration, CITE-seq

## Abstract

The retinal pigment epithelium (RPE) is a pigmented monolayer of cells beneath the neural retina that supports photoreceptor cell function essential for vision. Our study explores the diversity of adult human RPE subpopulations and associated implications for retinal biology. Employing cellular indexing of transcriptomes and epitopes by sequencing (CITE-seq), we identified distinct RPE cell subpopulations characterized by unique single-cell transcriptomic and surface protein signatures. Immunohistochemical analysis using CITE-seq markers demonstrated that different RPE subpopulations had previously unappreciated spatial patterns. Enrichment by CITE-seq surface marker selection revealed that different RPE subpopulations have distinct functions. By comparing native RPE cells isolated from the adult RPE layer to cultured RPE cells, we demonstrated that most RPE subpopulations were preserved during culture, a finding with relevance to an RPE cell product currently in clinical trial for treatment of non-exudative age-related macular degeneration. These findings deepen understanding of human RPE biology and provide valuable insights to optimize RPE-cell-based therapy.

## Highlights

• Native and cultured RPESC-RPE monolayers are heterogeneous in form and function
• CITE-seq analysis has revealed cell surface markers of each RPE subpopulation
• RPE subpopulations have spatially distinct localizations in the eye
• RPE subpopulations demonstrate unique functional capabilities

## Graphical abstract

**Figure:**
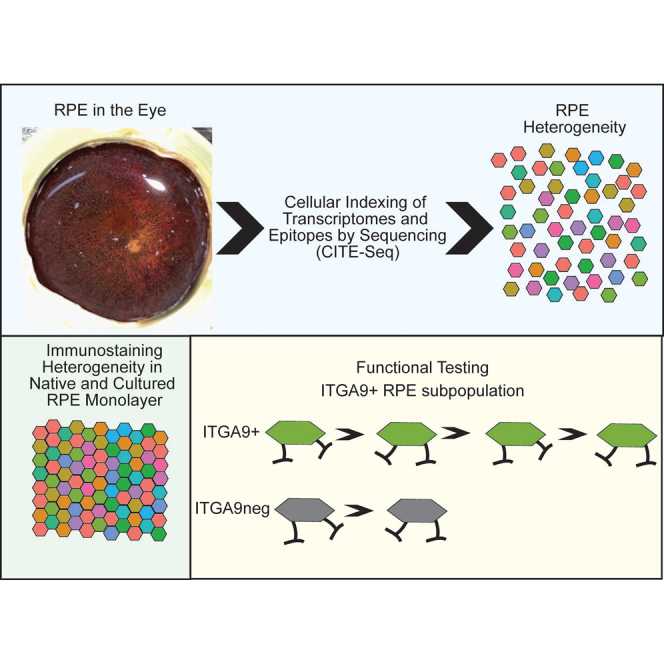

## Teaser

Farjood et al. has set the stage for characterizing RPE heterogeneity by using single-cell sequencing technology to identify cell surface markers compatible with isolating each subpopulation for further study. Using these markers, select RPE subpopulations are shown to have unique localization within the eye and to have distinct functional capabilities.

## Introduction

The retinal pigment epithelium (RPE) supports neural retinal photoreceptor cells by providing nourishment, phagocytosing photoreceptor cell outer segments, and other vital functions. The RPE has traditionally been considered a homogenous monolayer composed of one cell type, but more recently accumulating evidence points toward significant RPE cell diversity in morphology, gene expression, and function (Boulton et al., 1994; Farjood et al., 2023; Mullin et al., 2023; Ortolan et al., 2022; Xu et al., 2021). Understanding human RPE subpopulation diversity and associated functional and morphological specialization provides insights to improve understanding of retinal health and disease.

Transcriptional, proteomic, and morphological analysis of human RPE cells in macular versus peripheral anatomical retinal regions has revealed significant spatial heterogeneity within the RPE monolayer (Bhatia et al., 2016; Ortolan et al., 2022; Skeie and Mahajan, 2014; Voigt et al., 2019; Whitmore et al., 2014; Xu et al., 2021). RPE cells localized in the macular region are narrower and express lower levels of BEST1 and higher levels of complement proteins (Gao and Hollyfield, 1992; Skeie and Mahajan, 2014; Whitmore et al., 2014). Further evidence of RPE cell heterogeneity comes from our work demonstrating that a minor subpopulation of adult human RPE cells has the potential for extensive *in vitro* proliferation and self-renewal, defining the tissue-specific RPE stem cell (RPESC) (Saini et al., 2016; Salero et al., 2012).

Single-cell RNA sequencing (scRNA-seq) has improved our ability to detect cellular heterogeneity in retinal tissue (Li et al., 2023; Yan et al., 2020). This approach enabled the discovery of new ganglion cell subtypes in the mouse retina, (Tran et al., 2019) macroglial and photoreceptor cell subpopulations in the human retina (Lukowski et al., 2019; Menon et al., 2019), and the identification of diverse cell populations, including endothelial cells, macrophages, and RPE, from macular and peripheral regions of healthy and age-related macular degeneration (AMD) samples (Voigt et al., 2019). Most studies of RPE cell subpopulations utilized both choroidal and RPE cells, and the RPE cells identified were often considered a single homogeneous cell population among a multitude of diverse cell types (Collin et al., 2023; Huang et al., 2023; Monavarfeshani et al., 2023; Voigt et al., 2019). A few studies focused on the native RPE cells isolated directly from the human eye (Mullin et al., 2023; Xu et al., 2021) that revealed diverse RPE subpopulations with unique transcriptional and chromatin profiles (Mullin et al., 2023; Xu et al., 2021). These prior studies did not assess whether transcriptomic heterogeneity is associated with functional specialization nor whether the diverse RPE cells represented distinct subtypes or different labile cell states.

Cellular indexing of transcriptomes and epitopes by sequencing (CITE-seq) (Stoeckius et al., 2017) has significantly advanced our ability to uncover cellular heterogeneity by enabling the simultaneous measurement of surface protein expression and transcriptomic profiles within individual cells. This technique allows for deeper understanding of cellular diversity and identification of previously unrecognized subpopulations (England et al., 2020; Liu et al., 2023b; Pombo Antunes et al., 2021; Wu et al., 2021). The identification of surface markers enables cell subtypes to be enriched by sorting for functional studies. Here, we describe the first CITE-seq study focused on the adult human RPE acutely isolated from the adult human eye (native RPE) to better characterize RPE heterogeneity. This approach allowed us to comprehensively dissect RPE subpopulations and identify surface proteins that enabled RPE subpopulation enrichment and functional assessment. Multimodal analysis revealed a remarkable heterogeneity of 12 RPE cell identities, each with distinct gene and surface protein expression signatures, indicating that multiple, previously unrecognized cell subpopulations are present in the native RPE layer. We then studied the spatial distribution and morphological heterogeneity of these subpopulations within the human eye. We further demonstrated that RPE cells derived from native adult RPE after several weeks in culture recapitulate the subpopulations found in native RPE tissue. This supports the use of cultured adult RPE cells for *in vitro* modeling and as a cell source for replacing the RPE cells that are lost in retinal degenerative diseases.

## Results

### Native human RPE subpopulations have distinct surface protein expression

**Figure 1:**
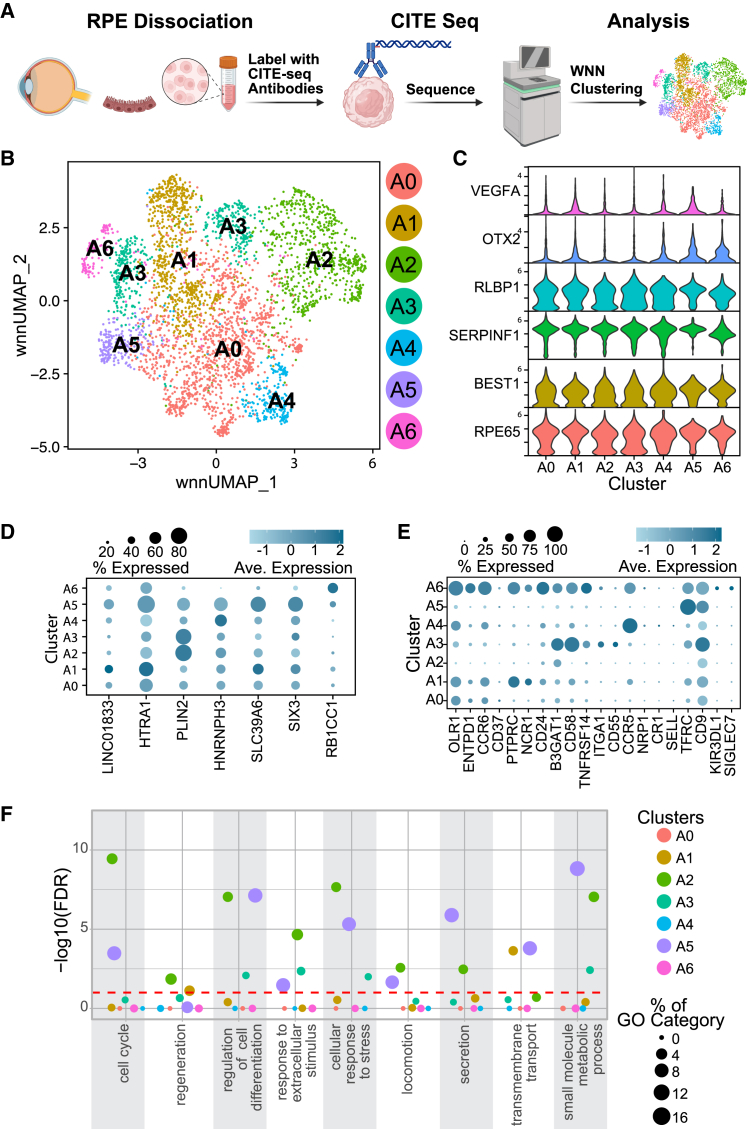
CITE-seq results revealed native RPE heterogeneity (*n* = 3, biological replicates) (A) Schematic of the CITE-seq study. (B) UMAP figure visualization of human RPE subpopulations clustered using WNN analysis. (C) Expression of RPE-specific markers in all clusters (log-normalized gene expression). (D) Gene markers of RPE clusters (z-normalized log1p gene expression). (E) Surface protein markers enriched in each cluster (z-normalized transformed ADT expression). (F) Selected parent GO categories from GO enrichment and semantic similarity analysis of RNA markers from each cluster.

The experimental approach is summarized in Figure 1A. Three human donor eyes (two males and one female) were obtained from registered eye banks, and RPE cells were dissociated for analysis. Utilizing a TotalSeq-A antibody cocktail, acutely isolated RPE cells were labeled with 156 uniquely barcoded antibodies, followed by single-cell library preparation using the ICELL8 system. After sequencing, transcript reads were mapped to the human genome (hg38), and antibody-derived tag (ADT) reads were mapped to an antibody/barcode sequence list. The ADT counts were preprocessed by arcsinh transformation and normalization. The final dataset consisted of 3,706 cells with an average read depth of 269,448 for transcriptomic data and 291,364 for the surfaceome profile. Dimensionality reduction, weighted nearest neighbor analysis (contributions of each modality can be found in Figure S1), and clustering were performed using the Seurat package in R.

After clustering, seven distinct RPE subpopulations were identified and designated A0 through A6 (Figure 1B). To verify the RPE identity of each cluster, we examined the expression of 171 genes designated to be RPE signature genes by bulk RNA sequencing (RNA-seq) analysis of acutely isolated RPE cells (Bennis et al., 2015; Strunnikova et al., 2010). One hundred forty of the signature genes were expressed by the primary RPE cells, with 95 shared between all clusters and each cluster expressing at least 101 signature genes (Figure 1C; Table S1), verifying the RPE identity of these subpopulations. Our results revealed unique gene and surface protein expression profiles or molecular signatures for each cluster (Figures 1D and 1E; Tables S2 and S3). For example, cluster A1 exhibited higher expression of HTRA1, which has been associated with an increased risk of AMD (Lin et al., 2018) and of the long noncoding RNA Linc01833, a biomarker of immune infiltration that is expressed in both cultured and native human RPE cells (Liu et al., 2023a; Postnikova et al., 2019). Clusters A2 and A3 showed higher expression of PLIN2, a gene responsive to oxidative stress and involved in the formation of lipid droplets by RPE cells, which is thought to contribute to drusen formation (Chuang et al., 2022; Grubaugh et al., 2023; Meyer et al., 2019). Cluster A4 displayed higher expression of HNRNPH3, an RNA splicing protein that is linked to an increased risk of AMD (Lin et al., 2018). Cluster A5 demonstrated enriched expression of a distinct set of genes, including SLC39A6, a zinc transporter, (Chowanadisai et al., 2008) and SIX3, a regulator of Wnt/β-catenin signaling that maintains neuro-retinal progenitors (Diacou et al., 2018). Cluster A6 exhibited higher levels of RB1CC1, essential for the autophagic activity of RPE cells (Yao et al., 2015).

We performed a Gene Ontology (GO) enrichment of the genes associated with each cluster that revealed specific functions were enriched (Figure 1F; Table S4). Clusters A0 and A4 did not demonstrate enrichment for any categories. Cluster A1 is enriched for metabolic processes and organization. Clusters A5 and A2 have the most enriched terms (937 and 598, respectively). While both clusters are enriched for the parent term “cell cycle,” cluster A2 is enriched for “negative regulation of cell cycle,” whereas A5 is enriched for the “positive regulation of cell cycle.” Cluster A5 is enriched for categories associated with “cell division,” whereas cluster A2 shows no such enrichment. These clusters also differ in categories related to “defense response” with cluster A2 showing enrichment in associated categories and cluster A5 demonstrating no enrichment. Like cluster A1, cluster A3 demonstrated enrichment for a variety of metabolic processes; however, A3 also showed enrichment for processes related to development and response to the environment. Finally, cluster A6 was primarily enriched for categories associated with “generation of precursor metabolites and energy.” Overall, the primary RPE clusters showed wide enrichment for different metabolic pathways; however, clusters A1, A2, A3, and A5 demonstrated enrichment for a broad array of non-metabolic functions.

The CITE-seq data identified distinct surface protein markers for each cluster (Figure 1E). While all clusters expressed RPE-specific markers (Figure 1C), cluster A1 exhibited higher expression of PTPRC (CD45), a hematopoietic marker known to be expressed in RPE cells (Limb et al., 1997), NCR1, a marker of natural killer cells (Moretta et al., 2001), and CD24, a neural progenitor cell marker that regulates autophagy (Lu et al., 2018; Robson et al., 2019; Sun et al., 2020). Clusters A2 and A3 had elevated expression of B3GAT1, a gene reduced in the RPE-choroid layers of AMD eyes (Newman et al., 2012; Wang et al., 2022). Cluster A3 had elevated levels of CD58, TNFRSF14, ITGA1, and CD55. CD58 and TNFRSF14 are involved in the immune response, while CD55 has a role in complement inhibition in RPE cells (Kim and Song, 2006; Ma et al., 2010; Yang et al., 2009). Cluster A3 expressed ITGA1, a gene involved in transforming growth factor beta (TGF-β)-induced epithelial-mesenchymal transition (EMT) (Gharibi et al., 2017). Cluster A4 showed significantly higher levels of the chemokine receptor, CCR5, which is linked to geographic atrophy (Krogh Nielsen et al., 2020; Nagineni et al., 2015). Cluster A5 was enriched with transferrin receptor (TFRC), associated with iron uptake (Hentze et al., 2004). Cluster A6 expressed a diverse range of surface proteins and uniquely exhibited high levels of the lectin SIGLEC7, an immune receptor, and KIR3DL1, which binds HLA (human leukocyte antigen) molecules (Boudreau et al., 2016; Stewart et al., 2024).

**Figure 2:**
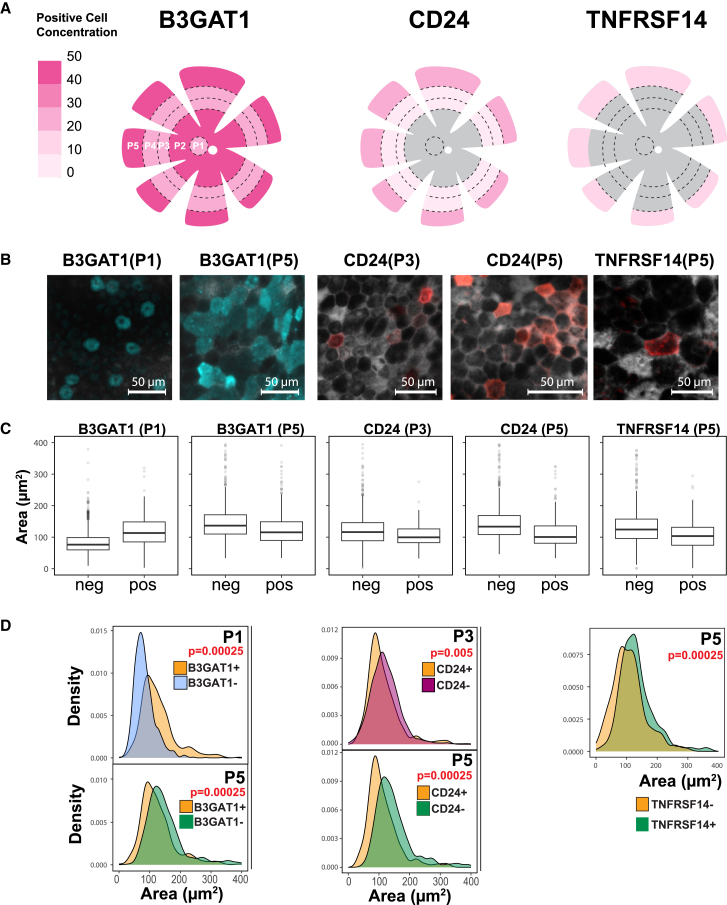
Immunofluorescence imaging of RPE flat mounts prepared from a human donor eye revealed subpopulations in human RPE monolayer (A) Schematics of human RPE showing spatial distribution of B3GAT1-, CD24-, and TNFRSF14-expressing cells in five concentric, morphologically different regions of the RPE described in a previous study (Ortolan et al., 2022). (B) Immunofluorescence images showing expression of B3GAT1, CD24, TNFRSF14, and ITGB3 in subpopulations of adult RPE cells in different regions of the human RPE tissue. Scale bars, 50 µm. (C) Boxplots showing the area of B3GAT1-, CD24-, and TNFRSF14-expressing cells in different regions of the human RPE (*n* = 3, biological replicates, independent experiments). (D) Density plots showing the cell area distribution for RPE subpopulations. B3GAT1-expressing cells in P1 had a larger area than B3GAT1 cells, while in P5 B3GAT1+ cells were smaller than the negative population. CD24- and TNFRSF14 expressing cells were found to be smaller than their non-expressing counterparts (*n* = 3, biological replicates, independent experiments).

Most of the surface markers detected in this CITE-seq analysis had not been previously reported in RPE cells. Hence, we evaluated their expression in human RPE tissue. We analyzed B3GAT1 (clusters A2, A3, and A6), CD24 (cluster A6), and TNFRSF14 (clusters A3 and A6). Immunostaining of human RPE tissue revealed positive expression for all three markers (Figure 2). We then investigated the spatial distribution of these markers in the human RPE layer. We used an established anatomic classification system that categorizes RPE cells into five concentric regions, ranging from the macular (P1) to periphery (P5) (Figures 2A and 2B) (Ortolan et al., 2022). Quantitative analysis revealed that B3GAT1 was expressed in cells across all five regions with higher frequency of expression in P2 and P5. CD24 expression was detected in regions P3, P4, and P5, while TNFRSF14 was exclusively localized to region P5. These results confirm the expression of these CITE-seq markers in human RPE *in situ* with distinct regional distributions.

Previous studies demonstrated a difference in RPE cell size based on location within the eye, with smaller cells in the P1 region and larger cells in the P5 region (Ortolan et al., 2022). We found B3GAT1-, CD24-, or TNFRSF14-positive cells in the different zones of the native tissue had different sizes (Figures 2C and 2D). B3GAT1+ cells within P1 or P5 show similar size distribution. However, B3GAT1+ cells were larger than their B3GAT1-neighbors in P1 but smaller than their B3GAT1-neighbors in P5. CD24^+^ cells showed a similar size distribution in both P3 and P5; however, CD24^+^ cells were smaller than the CD24^−^ cells. TNFRSF14+ cells in the P5 zone were smaller than their negative neighbors. These results underscore the unique distribution and morphology of cells bearing the three markers. Moreover, these observations indicate that the cluster A6 subpopulation (marked by TNFRSF14, B3GAT1, and CD24) is found in the P5 region and is smaller than the other cells in this region.

### Extensively cultured adult RPE cells maintain distinct subpopulation identities

**Figure 3:**
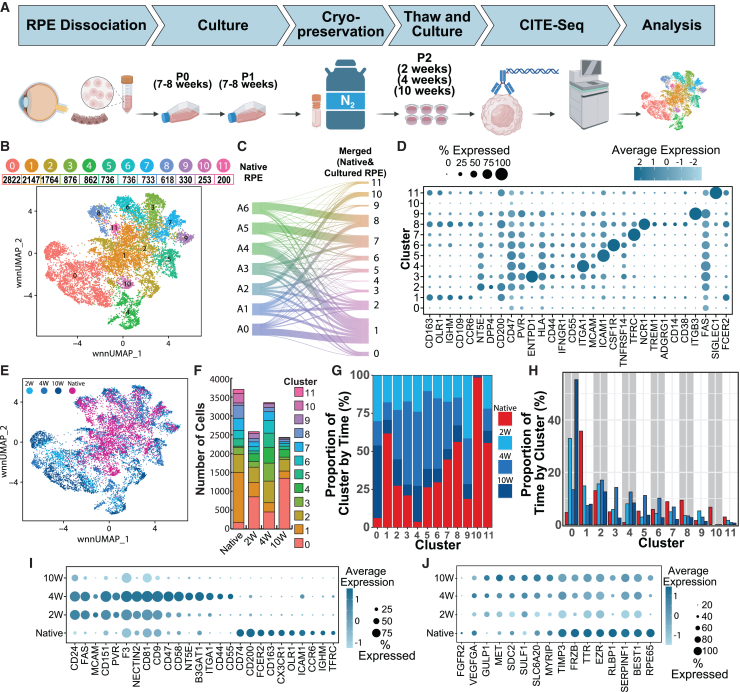
Analysis of CITE-seq results from both primary (*n* = 3, biological replicates) and adult cultured RPE (*n* = 3, biological replicates) reveals new RPE subpopulations (A) Schematic of the CITE-seq study. (B) UMAP figure visualization of combined native and cultured subpopulations clustered using WNN analysis. (C) River plot illustrating the contribution of the native RPE clusters to the integrated data clusters. (D) Surface protein markers of RPE clusters (z-normalized transformed ADT expression). (E) UMAP figure showing distribution of cells from different time points. (F) Proportional bar chart showing the distribution of RPE clusters across RPE source (native or cultured for 2,4,10 week). (G) Stacked bar plot illustrating the cell proportions by time points within RPE clusters. (H) Bar chart showing the portion of cells from each time within RPE clusters. (I) Surface protein markers enriched in different time points (z-normalized transformed ADT expression). (J) Changes in the expression of RPE-specific genes in native RPE and cultured RPE from 2W to 10W (z-normalized log1p gene expression).

To investigate whether adult RPE subpopulations are recapitulated in RPE cells derived from the proliferative RPE subpopulation, we performed CITE-seq experiments on passage 2 (P2) adult human RPE cells (Figure 3A). These cells had been cultured, passaged, frozen, then thawed to create P2 cells that were cultured for an additional 2, 4, or 10 weeks. After sequencing and QC, we obtained 2,586, 3,354, and 2431 cells in the 2W, 4W, and 10W cultures, respectively. The cultured cell CITE-seq data and the 3,706 primary RPE cell CITE-seq data were merged using canonical correlation analysis and clustered using the Seurat package in R. After merging the datasets, the average read depth was 221,231 per cell for the transcriptomic profiles and 272,903 per cell for the surfaceome profile. Due to the increased number of cells in the combined dataset, we were able to identify 12 RPE subpopulations, including ones not in the primary cell data alone (Figure 3B).

Each of the 12 RPE cell clusters exhibited expression of at least 110 RPE signature genes, with 91 genes shared across all clusters and 140 RPE signature genes in the combined dataset. Clusters 7, 8, and 11 displayed the highest number of RPE signature genes (129, 128, and 126, respectively), while cluster 4 exhibited the lowest number (Table S5). This analysis verified the RPE identity of all native and cultured populations. Most of the clusters found in primary RPE cells were preserved in the combined dataset, showing preservation native RPE subpopulations in cultured RPE preparations (Figure 3C), albeit with differences in the individual subpopulation contributions to the whole. Analyzing the merged CITE-seq data identified unique gene and surface markers for each cluster (Tables S6 and S7) similar to that found in native RPE subpopulations (Figures 1E and 3D).

We quantified the contribution of each RPE source (native RPE and P2 RPE cells cultured for 2, 4, or 10 weeks) to each cluster (Figures 3E–3H). Except for cluster 10, which contained mainly native cells, all clusters contained cells from both native and cultured RPE preparations, although the percentages varied. The predominant composition of clusters 1, 7, 8, 10, and 11 are native RPE cells, while clusters 0 and 4 are mainly comprised of cultured RPE cells (95%; Figure 3G). Similar percentages of cells were present regardless of time in clusters 2, 7, and 5 (Figure 3H). Overall, the cultured and native RPE demonstrate a similar subpopulation composition.

### RPE exhibits unique surfaceome and morphological profiles between subpopulations

Cultured and native RPE showed a distinct surface protein signature by CITE-seq (Figure 3I). Forty-three of the probed surface proteins showed differential expression across native and P2 cultured cells (Table S8). Both native and cultured RPE cells had a higher expression of different subsets of proteins when compared (Figure 3I). RPE-4W cells had a unique gene and surface protein signature (Tables S8 and S9) with elevated expression of CD47, CD58, NT5E, B3GAT1, ITGA1, CD44, CD55, and TNFRSF14 (Figure 3I). Interestingly, the expression levels of all enriched RPE-4W markers decreased at 10 weeks as the cells matured, reaching levels like those observed in primary cells (Figure 3I). We also observed an increase in the expression of RPE signature genes from 2 to 10 weeks in cultured RPE cells (Figure 3J), further suggesting that *in vitro* maturation of RPE cells occurs during time in culture. We next identified surface markers that could be used to enrich RPE subpopulations, for example, using magnetic or FACS-based isolation methods (Figure 3D). For instance, CSF1R or ITGB3 can be used to isolate cluster 6 or 9 subpopulations, respectively (Figure S2).

**Figure 4:**
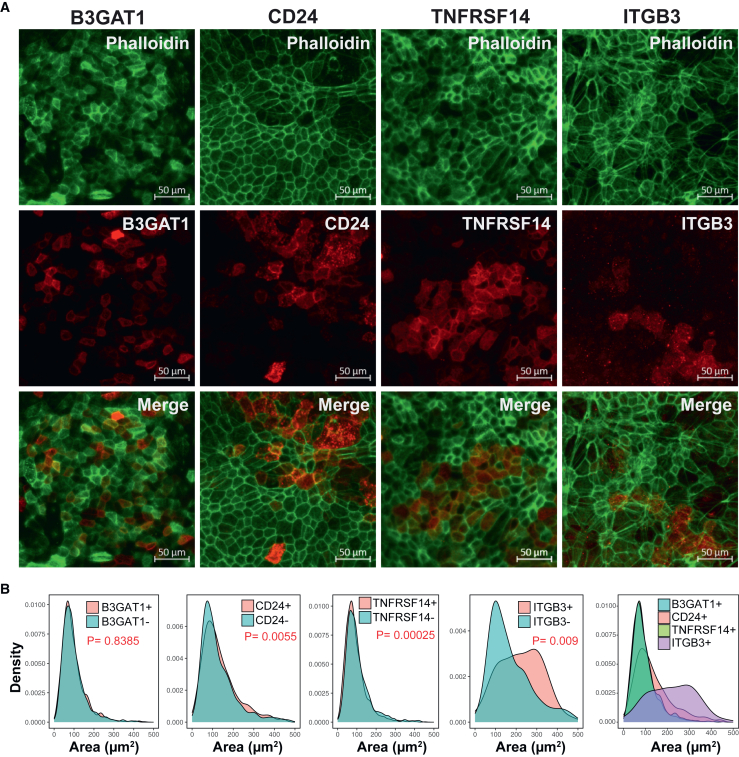
Immunofluorescence imaging of RPE-4W cultures shows expression of cultured RPE surface markers (A) Subpopulations of RPE cells were detected in RPE-4W cells expressing B3GAT1, CD24, and TNFRSF14 surface proteins that were observed to be upregulated in RPE-4W cells according to CITE-seq analysis (Figure 1F) (*n* = 3, biological replicates, concurrent experiments). ITGB3, a marker of cluster 9, was also found to be expressed in RPE-4W cultures. Scale bar, 50 µm. (B) Morphological examination of cells expressing B3GAT1, CD24, and ITGB3 revealed distinct characteristics. CD24^+^ and ITGB3+ cells exhibited larger cellular areas, whereas TNFRSF14+ cells displayed a smaller area compared to their negative counterparts. ITGB3-positive cells demonstrated the largest cellular area among the four subpopulations.

Given our finding of surface markers that distinguish different clusters, our next goal was to confirm these findings through immunofluorescence microscopy of cultured RPE cells. Immunostaining was performed on P2-cultured RPE-4W cells, targeting B3GAT1, CD24, and TNFRSF14, and the cluster 9 marker, ITGB3 (Figure 4A). CITE-seq showed B3GAT1, CD24, TNFRSF14, and ITGB3 were expressed in 77.8%, 91.6%, 50.7%, and 4.7% of the RPE-4W cells, respectively. Analysis revealed similar proportions of positive cells for TNFRSF14 and ITGB3 populations (55.6% and 6.8%, respectively), with fewer B3GAT1 and CD24-expressing cells than predicted by the CITE-seq data (36% and 52%, respectively), possibly due to a lower sensitivity of immunocytochemistry (Stoeckius et al., 2017).

**Figure 5:**
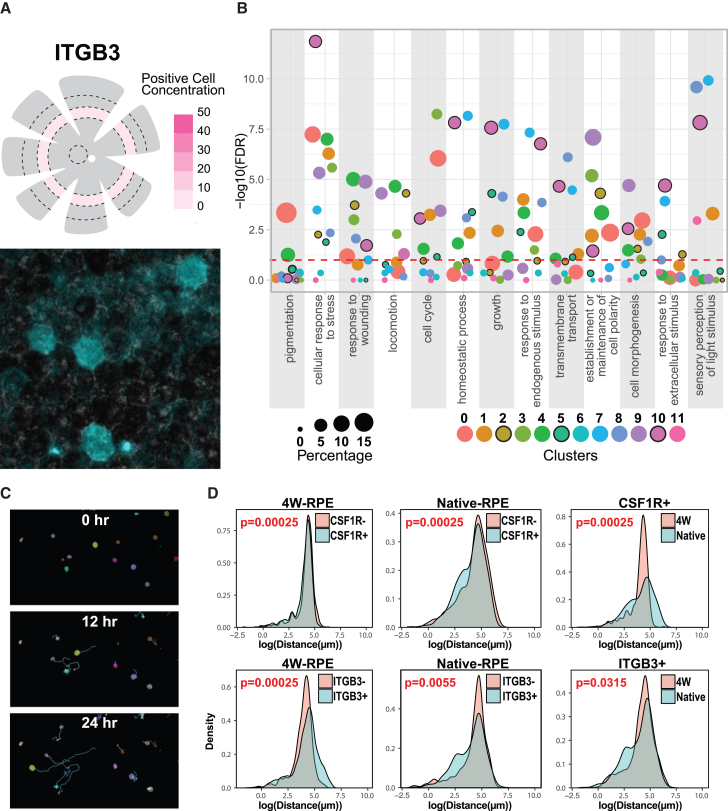
Gene Ontology (GO) enrichment analysis reveals RPE subpopulation functional specialization (A) Expression of ITGB3 in human RPE tissue was confirmed through immunofluorescence imaging of a human RPE flat mount. The spatial distribution of ITGB3+ cells was analyzed as described in Figure 2. (*n* = 3, biological replicates, independent experiments) (B) GO enrichment and semantic similarity analysis of RPE subpopulations. The dot size reflects the percentage of genes in each category expressed by the cluster, and the *y* axis represents the log of the false discovery rate (FDR) for the enrichment of each category. (C) Representative images of automated cell tracking results showing cells with low- and high-migratory capacity. (D) Density plots showing the distribution of migration distance for cells expressing markers of clusters 6 and 9, CSF1R and ITGB3, respectively, in native RPE and RPE-4W cells. CSF1R-expressing cells had a lower migration distance compared to CSF1R− cells in both native and RPE-4W cultures. ITGB3+ cells of the native and RPE-4W cultures traveled a longer distance compared to their negative counterparts. (*n* = 3, biological replicates, independent experiments).

Morphological analysis revealed that CD24^+^ and ITGB3+ cells were larger, while TNFRSF14+ cells were smaller than their negative counterparts (Figure 4B). In RPE-4W cultures, B3GAT1+ and TNFRSF14+ cells were smaller compared to native RPE cells, whereas CD24^+^ and ITGB3+ cells were larger. Expression frequency comparisons showed that B3GAT1 expression increased from 36% in native RPE to 78% in RPE-4W cells, CD24 increased from 23% to 92%, and TNFRSF14 increased from 17% to 51%. ITGB3 expression remained stable, with 4% in native RPE and 4.7% in RPE-4W cells. Immunofluorescence imaging revealed a small ITGB3+ subpopulation (∼1.6%) within the P3 region of native RPE tissue (Figure 5A).

### Functional diversity among RPE subpopulations

To gain insight into the functional diversity among molecularly distinct RPE subpopulations, we identified the gene expression associated with each cluster using Seurat’s FindAllMarkers function (Table S6) and performed GO pathway enrichment analysis utilizing the identified genes (Figure 5B). We employed a semantic similarity approach provided by the “screp” package (https://github.com/neural-stem-cell-institute/screp) in R (Table S10). Our analysis revealed significant diversity in pathway enrichment across RPE subpopulations. Clusters 0, 3, and 9 expressed more in cultured cells exhibited highly significant enrichment for pathways associated with cell-cycle regulation and cell division. Despite the lower expression levels of RPE-specific markers within these subpopulations, likely indicative of a less mature differentiation state, the subpopulations remained enriched for pathways specific to fundamental RPE functions, such as those involved in pigmentation and cell polarity (Figure 5B).

Clusters 1 and 10 exhibited enrichment in pathways associated with metabolic processes, stress response, and homeostasis pathways. Clusters 2 and 3 had enrichment for cell cycle, migration, cytoskeleton organization, and response to stress. Cluster 5 was enriched for pathways “response to endogenous stimulation” and “homeostatic processes.” Clusters 7, 8, and 11 showed enrichment for “sensory perception of light stimulus” and “vascular process in circulatory system.” Cluster 9, marked by expression of ITGB3, exhibited predominant and substantial enrichment in pathways associated with cell morphogenesis, migration, locomotion, and regulation of organelle organization, suggesting a potential emphasis on cell motility within this cluster. ITGB3+ RPE cell motility was confirmed using an *in vitro* functional assay.

### ITGB3+ cells are more migratory than CSFR+ RPE cells

Due to autofluorescence of native RPE cells, we employed magnetic-activated cell sorting (MACS) to separate ITGB3+ (cluster 9) and CSF1R+ (cluster 6) cells in native RPE and RPE-4W cells. Each subpopulation was separately cultured under time-lapse microscopy over 24 h. Time-lapse videos were analyzed to quantify migration using the Bayesian Tracker (btrack) package in Python (Figure 5C, supplementary information 1, Video S1) (Ulicna et al., 2021).

Video S1. Example cell tracking results using the btrack package

CSF1R+ cells exhibited a slightly lower migratory capacity and a slower migratory rate compared to CSF1R− cells in both native RPE and cultured RPE-4W. However, native CSF1R+ RPE cells migrated longer distances compared to their cultured counterparts (Figure 5D). The motility of ITGB3+ cells in both native and RPE-4W showed a bimodal distribution, indicating two distinct migratory potentials within the cluster 9 subpopulation. The larger population demonstrated high motility, with a peak migration distance of approximately 150 μm, while the smaller population exhibited lower motility, with a peak migration distance of around 12 μm. Hence the cluster 9 cells, which were enriched for markers of cell motility, demonstrated increased migration. These data for the first time demonstrates a specialized cellular function associated with a distinct isolated RPE subpopulation.

## Discussion

RPE cell heterogeneity is increasingly recognized as a critical aspect of retinal physiology and pathology. Recent studies have identified multiple RPE subpopulations by transcriptome analysis, but a full understanding of the functionally distinct RPE subpopulations is still lacking (Collin et al., 2023; Huang et al., 2023; Mullin et al., 2023; Ortolan et al., 2022; Voigt et al., 2019; Xu et al., 2021). Our approach builds on previous work identifying cell surface proteins along with the transcriptional signatures of individual RPE cells. This enables us to identify and enrich different subpopulations using surface marker expression to assess morphological and functional specialization. We established seven subpopulations of native RPE cells with diverse morphology and function that are distributed in different regions of the RPE layer. Notably, these differences are propagated over long-term culture, consistent with the RPE cells being true subpopulations rather than labile states. Our findings highlight the value of cultured adult RPESC-RPE cells as an *in vitro* model of the native RPE tissue and as a source of cells to replace native RPE upon transplantation.

Prior studies demonstrated that RPE cells show morphological differences in the diseased state, during aging, and across different regions of the eye (Ortolan et al., 2022; Rashid et al., 2016). Previous studies associated variations in cell size with differences in mitochondrial function and metabolic activity (Miettinen and Bjorklund, 2017; Seel et al., 2023). Our morphological analysis revealed size differences among subpopulations in both native and cultured RPE cells. We identified an ITGB3+ subpopulation (cluster 9) characterized by a larger cell size and greater migratory capacity compared to ITGB3− cells. Notably, the migratory capacity of ITGB3+ cells remained consistent from native to long-term cultured RPE, demonstrating the stability of this functional specialization *in vitro*.

Our mapping of diverse RPE subpopulations to different regions of the RPE layer, combined with previous studies showing heterogeneous responses of RPE cells to disease processes (Ach et al., 2014; Ortolan et al., 2022; Rashid et al., 2016), suggests that specific RPE subpopulations have different susceptibilities to retinal disorders. We found that in native RPE subpopulations cluster A1 expresses multiple AMD-related genes, cluster A3 expresses genes involved in lipid droplet accumulation in the RPE (Hara et al., 2023), and cluster A6 expresses genes involved in autophagy (Yao et al., 2015). Clusters A6 and A3 are both associated with the outer RPE layer. We speculate that defects in autophagy and increases in lipid accumulation due to changes in the A3 and A6 subpopulations in the central retina may be due to subpopulation-specific pathologies that contribute to AMD pathogenesis (Wei et al., 2023). Hence, RPE cell subpopulation distribution may explain why retinal disease progression occurs at variable rates in different regions of the RPE layer, such as the macular region.

Promising vision improvements have been obtained in preclinical and early clinical studies using human RPE cell products to replace degenerated RPE tissue (Cho et al., 2019; Kajita et al., 2024; Liu et al., 2021; Mandai et al., 2017; Mehat et al., 2018; Qiu, 2019; Sharma et al., 2019; Song et al., 2015; Takagi et al., 2019; Thomas et al., 2016). While several studies have used pluripotent stem cells (Cho et al., 2019; Kajita et al., 2024; Mandai et al., 2017; Mehat et al., 2018; Qiu, 2019; Sharma et al., 2019; Song et al., 2015; Takagi et al., 2019; Thomas et al., 2016) as RPE sources, our previous work demonstrated that adult RPESC can serve as a practical source of RPE progeny for both modeling and RPE cell replacement therapy (Blenkinsop et al., 2015; Boles et al., 2020; Davis et al., 2017; Rabin et al., 2013). In this study, we show that cultured adult RPESCs recapitulate the major RPE subpopulations observed in acutely isolated, native RPE cells, with only minor differences in composition and gene expression. Our current findings support the use of cultured RPESC-RPE cells to replace RPE cells lost in diseases such as AMD. Moreover, these findings underscore the value of RPESC-RPE to model the native RPE layer *in vitro* (Fortress et al., 2023).

While the surface expression profiles of cultured and native RPE were similar, native RPE showed enrichment of immune-associated surface markers, including CD74 (regulates macrophage activation) (Schroder, 2016), CD200 (inhibiting microglial activation) (Zhang et al., 2011), and CX3CR1 (role in immune cell migration) (Lee et al., 2018). In addition to their role on immune cells, CD74, CD200, and CX3CR1 can also be expressed by endothelial cells to interact with immune cells. The expression of these markers indicates dynamic immune cross-talk in native RPE, contrasting with cultured cells where corresponding immune interactions are not present. It is possible that following transplantation, cultured RPE may adopt these native immune-related expression patterns upon integration with the host tissue.

In conclusion, our study provides a comprehensive characterization of adult human RPE subpopulations within native tissue and in culture. This characterization deepens our understanding of RPE biology and pathology and underscores the utility of RPESC-RPE.

## Methods

### Experimental model and subject details

CITE-seq was performed on native RPE cells from three adult human donor eyes and three cultured adult RPE cell lines at passage 2. Donor eyes were obtained from approved eye banks with consent for research use. The donor details are listed in Table S11. RPE cells were dissociated from donor tissues, and CITE-seq was performed on native and cultured cells. Cultured cells were examined for cobblestone morphology prior to use.

### Method details

#### Eye dissection and RPE dissociation

Human eyes were cut at the ora serrata, and the RPE layer was exposed by removing the anterior segment, the vitreous, and retina. Whole RPE dissection was performed with care taken to avoid the edge of the posterior eyecup or to puncture Bruch’s membrane (Blenkinsop et al., 2013). RPE cells were dissociated by incubating the RPE with collagenase IV (Worthington Biochemicals) at 37°C in a humidified incubator. Cells were either immediately utilized or passaged as RPESC-RPE (additional details in supplemental methods).

#### CITE-seq

After dissociation of adult RPE cells from human donor eyes or cultured cells from transwell inserts, the resulting single RPE cells were tagged with the TotalSeq-A human universal cocktail, V1.0 (BioLegend), containing 154 unique cell surface antigens and 9 isoform control antibodies. Cells were then washed three times with DPBS and stained with 1 μL SYTO64 dye (Invitrogen, Carlsbad, CA) in 1mL DPBS for 20 min at room temperature, then washed twice in fresh DPBS. Next, cells were diluted to 25,000 cells/mL and dispensed into ICELL8 3′ DE chips (Takara Bio, CA) using an MSND device (Takara Bio). Cell dispensing and in-chip reverse-transcription PCR were performed using a 3′ DE Chip and Reagent kit (Takara Bio) according to the manufacturer’s instructions. Libraries were prepared using our in-house protocols, then sequenced on a NovaSeq 6000 high-output flow cell, generating 2 × 150-bp read lengths (GeneWiz). Raw reads were processed and mapped to human hg38 genome and count matrices generated for further analysis (additional details in supplemental methods). Full analysis code is available at https://github.com/neural-stem-cell-institute/RPE-CITE-Seq.

#### RPE flat mount preparation and immunofluorescence staining

Human donor eyes were dissected as described above. After removing the retina, the eyecup was filled with 4% paraformaldehyde to fix the RPE-choroid for 1 h at room temperature. RPE-choroid was flattened by cutting the RPE-choroid-sclera from the edge toward the optic nerve. Flat mount pieces containing peripheral and central RPE were moved to glass slides and immunostained with conjugated primary antibodies diluted in 2% BSA according to the manufacturer instructions. Tissues were then washed with DBPS three times and covered with glass coverslips using Fluoromount-G (Thermo Fisher) mounting media and imaged using a fluorescence microscope.

#### Cell sorting

Magnetic activated cell sorting (MACS) and florescence-activated cell sorting (FACS) were performed for positive selection of RPE subpopulations. For MACS sorting, RPE cells were immunostained using phycoerythrin (PE)- or allophycocyanin (APC)-conjugated antibodies, and MACS anti-PE of anti-APC magnetic microbeads were used to capture positively labeled cells using MACS MS Columns (Miltenyi Biotec). Briefly, cells were incubated with PE- or APC-conjugated primary antibodies for 20 min, rinsed twice with DPBS+2% BSA and captured in MS columns attached to an MACS separator magnet (Miltenyi Biotec). Unlabeled cells were collected by washing the column twice with RPE medium containing 10% FBS, and labeled cells were released by removing the column from the magnetic stand and washing the column with fresh RPE medium with 10% FBS. Flow cytometry and FACS sorting were performed using an ARIA I system (BD Biosciences).

#### Time-lapse imaging and cell migration analysis

After cell sorting, labeled and unlabeled cells were cultured in 12-well plates at ∼10,000 cells per well. Time-lapse microscopy was performed on cultured cells using a Zeiss Axio Observer Z1 microscope equipped with a humidified incubator at 37°C and 5% CO2. Time-lapse videos were analyzed in Python (V 3.12.2) using pyclesperanto and napari-pyclesperanto-assistant plugin for segmentation, and btrack for cell tracking. The analysis of tracking data and figure generation was performed in R (V 4.3.2).

#### Image processing

Morphological analysis was performed by analyzing phase contrast and phalloidin staining images from human RPE or cultured RPE. Images were converted to binary, and cell-cell junctions were detected using the Ridge Detection plugin for ImageJ (https://ieeexplore.ieee.org/abstract/document/659930). Object identification and morphological analysis were performed using the EBImage package in R (Pau et al., 2010).

#### Quantification and statistical analysis

Analysis of single-cell transcriptomic data was carried out in R utilizing the following packages: Seurat (V4 and V5), ggplot2, hypeR, rrvgo, GOfuncR, igraph, and riverplot packages. Differences in the distribution of cell area in morphological analysis and migration distance in cell tracking were analyzed with a two-sample test based on Wasserstein’s distance using the two-sample package (V 2.0.1) in R.

## Resource availability

### Lead contact

Further information and request for resources and reagents should be directed to the lead contact, Nathan Boles (nathanboles@neuralsci.org).

### Materials availability

This study did not generate new reagents.

### Data and code availability

The accession number for the raw single-cell RNA-seq (scRNA-seq) data reported in this paper is GEO: GSE244572 (GEO: https://www.ncbi.nlm.nih.gov/geo/query/acc.cgi?acc=GSE244572). Code written for this study is available at GitHub (GitHub: https://github.com/neural-stem-cell-institute/RPE-CITE-Seq). FAIR and CARE data management principles were followed.

## Acknowledgments

This research was supported by the 10.13039/100000002National Institutes of Health (R01EY032138 to S.T. and N.C.B., R01EY029281 to J.H.S. and N.C.B., and U01EY030581 to J.H.S.). Additional funding was provided by Luxa Biotechnology. We also thank NeuraCell (Albany, NY) for assistance with FACS and flow cytometry experiments.

## Author contributions

Conceptualization, S.T., J.S., N.C.B., and F.F.; methodology, F.F., T.B., T.K., B.A., S.T., J.S., and N.C.B.; software, F.F., S.S., T.K., and N.C.B; validation, F.F., S.N., and N.C.B; formal analysis, F.F., S.N., J.B., S.S., J.W., and N.C.B.; investigation, F.F., S.N., T.B., J.B., S.S., J.W., and N.C.B.; resources, S.T., J.S., and N.C.B.; writing—original draft, F.F. and N.C.B.; writing—review & editing, F.F., S.T., J.S., and N.C.B.; visualization, F.F. and N.C.B.; supervision, S.T., J.S., and N.C.B.; project administration, J.S. and N.C.B.; funding acquisition, J.S. and N.C.B.

## Declaration of interests

N.C.B., F.F., B.A., S.T., and J.S. are listed as inventors on a provisional patent related to this work. The authors declare no other competing interests.

## Supplemental information

Data S1. Tables S1–S11

Document S3. Article plus supplemental information

## References

[bib1] Ach T., Huisingh C., McGwin G., Messinger J.D., Zhang T., Bentley M.J., Gutierrez D.B., Ablonczy Z., Smith R.T., Sloan K.R., Curcio C.A. (2014). Quantitative autofluorescence and cell density maps of the human retinal pigment epithelium. Investig. Ophthalmol. Vis. Sci..

[bib2] Bennis A., Gorgels T.G.M.F., Ten Brink J.B., van der Spek P.J., Bossers K., Heine V.M., Bergen A.A. (2015). Comparison of Mouse and Human Retinal Pigment Epithelium Gene Expression Profiles: Potential Implications for Age-Related Macular Degeneration. PLoS One.

[bib3] Bhatia S.K., Rashid A., Chrenek M.A., Zhang Q., Bruce B.B., Klein M., Boatright J.H., Jiang Y., Grossniklaus H.E., Nickerson J.M. (2016). Analysis of RPE morphometry in human eyes. Mol. Vis..

[bib4] Blenkinsop T.A., Saini J.S., Maminishkis A., Bharti K., Wan Q., Banzon T., Lotfi M., Davis J., Singh D., Rizzolo L.J. (2015). Human Adult Retinal Pigment Epithelial Stem Cell-Derived RPE Monolayers Exhibit Key Physiological Characteristics of Native Tissue. Investig. Ophthalmol. Vis. Sci..

[bib5] Blenkinsop T.A., Salero E., Stern J.H., Temple S. (2013). The culture and maintenance of functional retinal pigment epithelial monolayers from adult human eye. Methods Mol. Biol..

[bib6] Boles N.C., Fernandes M., Swigut T., Srinivasan R., Schiff L., Rada-Iglesias A., Wang Q., Saini J.S., Kiehl T., Stern J.H. (2020). Epigenomic and Transcriptomic Changes During Human RPE EMT in a Stem Cell Model of Epiretinal Membrane Pathogenesis and Prevention by Nicotinamide. Stem Cell Rep..

[bib7] Boudreau J.E., Mulrooney T.J., Le Luduec J.B., Barker E., Hsu K.C. (2016). KIR3DL1 and HLA-B Density and Binding Calibrate NK Education and Response to HIV. J. Immunol..

[bib8] Boulton M., Moriarty P., Jarvis-Evans J., Marcyniuk B. (1994). Regional variation and age-related changes of lysosomal enzymes in the human retinal pigment epithelium. Br. J. Ophthalmol..

[bib9] Cho S.M., Lee J., Lee H.B., Choi H.J., Ryu J.E., Lee H.J., Park H.K., Lee M.J., Lee J., Lee H.J. (2019). Subretinal transplantation of human embryonic stem cell-derived retinal pigment epithelium (MA09-hRPE): A safety and tolerability evaluation in minipigs. Regul. Toxicol. Pharmacol..

[bib10] Chowanadisai W., Lönnerdal B., Kelleher S.L. (2008). Zip6 (LIV-1) regulates zinc uptake in neuroblastoma cells under resting but not depolarizing conditions. Brain Res..

[bib11] Chuang J.Z., Yang N., Nakajima N., Otsu W., Fu C., Yang H.H., Lee M.P., Akbar A.F., Badea T.C., Guo Z. (2022). Retinal pigment epithelium-specific CLIC4 mutant is a mouse model of dry age-related macular degeneration. Nat. Commun..

[bib12] Collin J., Hasoon M.S.R., Zerti D., Hammadi S., Dorgau B., Clarke L., Steel D., Hussain R., Coxhead J., Lisgo S. (2023). Single-cell RNA sequencing reveals transcriptional changes of human choroidal and retinal pigment epithelium cells during fetal development, in healthy adult and intermediate age-related macular degeneration. Hum. Mol. Genet..

[bib13] Davis R.J., Alam N.M., Zhao C., Müller C., Saini J.S., Blenkinsop T.A., Mazzoni F., Campbell M., Borden S.M., Charniga C.J. (2017). The Developmental Stage of Adult Human Stem Cell-Derived Retinal Pigment Epithelium Cells Influences Transplant Efficacy for Vision Rescue. Stem Cell Rep..

[bib14] Diacou R., Zhao Y., Zheng D., Cvekl A., Liu W. (2018). Six3 and Six6 Are Jointly Required for the Maintenance of Multipotent Retinal Progenitors through Both Positive and Negative Regulation. Cell Rep..

[bib15] England A.R., Chaney C.P., Das A., Patel M., Malewska A., Armendariz D., Hon G.C., Strand D.W., Drake K.A., Carroll T.J. (2020). Identification and characterization of cellular heterogeneity within the developing renal interstitium. Development.

[bib16] Farjood F., Manos J.D., Wang Y., Williams A.L., Zhao C., Borden S., Alam N., Prusky G., Temple S., Stern J.H., Boles N.C. (2023). Identifying biomarkers of heterogeneity and transplantation efficacy in retinal pigment epithelial cells. J. Exp. Med..

[bib17] Fortress A.M., Miyagishima K.J., Reed A.A., Temple S., Clegg D.O., Tucker B.A., Blenkinsop T.A., Harb G., Greenwell T.N., Ludwig T.E., Bharti K. (2023). Stem cell sources and characterization in the development of cell-based products for treating retinal disease: An NEI Town Hall report. Stem Cell Res. Ther..

[bib18] Gao H., Hollyfield J.G. (1992). Aging of the human retina. Differential loss of neurons and retinal pigment epithelial cells. Investig. Ophthalmol. Vis. Sci..

[bib19] Gharibi A., La Kim S., Molnar J., Brambilla D., Adamian Y., Hoover M., Hong J., Lin J., Wolfenden L., Kelber J.A. (2017). ITGA1 is a pre-malignant biomarker that promotes therapy resistance and metastatic potential in pancreatic cancer. Sci. Rep..

[bib20] Grubaugh C.R., Dhingra A., Prakash B., Montenegro D., Sparrow J.R., Daniele L.L., Curcio C.A., Bell B.A., Hussain M.M., Boesze-Battaglia K. (2023). Microsomal triglyceride transfer protein is necessary to maintain lipid homeostasis and retinal function. bioRxiv.

[bib21] Hara M., Wu W., Malechka V.V., Takahashi Y., Ma J.X., Moiseyev G. (2023). PNPLA2 mobilizes retinyl esters from retinosomes and promotes the generation of 11-cis-retinal in the visual cycle. Cell Rep..

[bib22] Hentze M.W., Muckenthaler M.U., Andrews N.C. (2004). Balancing acts: molecular control of mammalian iron metabolism. Cell.

[bib23] Huang L., Ye L., Li R., Zhang S., Qu C., Li S., Li J., Yang M., Wu B., Chen R. (2023). Dynamic human retinal pigment epithelium (RPE) and choroid architecture based on single-cell transcriptomic landscape analysis. Genes Dis..

[bib24] Kajita K., Nishida M., Kurimoto Y., Yokota S., Sugita S., Semba T., Shirae S., Hayashi N., Ozaki A., Miura Y. (2024). Graft cell expansion from hiPSC-RPE strip after transplantation in primate eyes with or without RPE damage. Sci. Rep..

[bib25] Kim D.D., Song W.C. (2006). Membrane complement regulatory proteins. Clin. Immunol..

[bib26] Krogh Nielsen M., Subhi Y., Molbech C.R., Falk M.K., Nissen M.H., Sørensen T.L. (2020). Chemokine Profile and the Alterations in CCR5-CCL5 Axis in Geographic Atrophy Secondary to Age-Related Macular Degeneration. Investig. Ophthalmol. Vis. Sci..

[bib27] Lee M., Lee Y., Song J., Lee J., Chang S.Y. (2018). Tissue-specific Role of CX(3)CR1 Expressing Immune Cells and Their Relationships with Human Disease. Immune Netw..

[bib28] Li J., Wang J., Ibarra I.L., Cheng X., Luecken M.D., Lu J., Monavarfeshani A., Yan W., Zheng Y., Zuo Z. (2023). Integrated multi-omics single cell atlas of the human retina. Preprint at Res Sq.

[bib29] Limb G.A., Cole C.J., Earley O., Hollifield R.D., Russell W., Stanford M.R. (1997). Expression of hematopoietic cell markers by retinal pigment epithelial cells. Curr. Eye Res..

[bib30] Lin M.K., Yang J., Hsu C.W., Gore A., Bassuk A.G., Brown L.M., Colligan R., Sengillo J.D., Mahajan V.B., Tsang S.H. (2018). HTRA1, an age-related macular degeneration protease, processes extracellular matrix proteins EFEMP1 and TSP1. Aging Cell.

[bib31] Liu W., Wan Q., Zhou E., He P., Tang L. (2023). LncRNA LINC01833 is a Prognostic Biomarker and Correlates with Immune Infiltrates in Patients with Lung Adenocarcinoma by Integrated Bioinformatics Analysis. J. Oncol..

[bib32] Liu Y., DiStasio M., Su G., Asashima H., Enninful A., Qin X., Deng Y., Nam J., Gao F., Bordignon P. (2023). High-plex protein and whole transcriptome co-mapping at cellular resolution with spatial CITE-seq. Nat. Biotechnol..

[bib33] Liu Z., Parikh B.H., Tan Q.S.W., Wong D.S.L., Ong K.H., Yu W., Seah I., Holder G.E., Hunziker W., Tan G.S.W. (2021). Surgical Transplantation of Human RPE Stem Cell-Derived RPE Monolayers into Non-Human Primates with Immunosuppression. Stem Cell Rep..

[bib34] Lu S., Yao Y., Xu G., Zhou C., Zhang Y., Sun J., Jiang R., Shao Q., Chen Y. (2018). CD24 regulates sorafenib resistance via activating autophagy in hepatocellular carcinoma. Cell Death Dis..

[bib35] Lukowski S.W., Lo C.Y., Sharov A.A., Nguyen Q., Fang L., Hung S.S., Zhu L., Zhang T., Grünert U., Nguyen T. (2019). A single-cell transcriptome atlas of the adult human retina. EMBO J..

[bib36] Ma K.N., Cashman S.M., Sweigard J.H., Kumar-Singh R. (2010). Decay accelerating factor (CD55)-mediated attenuation of complement: therapeutic implications for age-related macular degeneration. Investig. Ophthalmol. Vis. Sci..

[bib37] Mandai M., Watanabe A., Kurimoto Y., Hirami Y., Morinaga C., Daimon T., Fujihara M., Akimaru H., Sakai N., Shibata Y. (2017). Autologous Induced Stem-Cell-Derived Retinal Cells for Macular Degeneration. N. Engl. J. Med..

[bib38] Mehat M.S., Sundaram V., Ripamonti C., Robson A.G., Smith A.J., Borooah S., Robinson M., Rosenthal A.N., Innes W., Weleber R.G. (2018). Transplantation of Human Embryonic Stem Cell-Derived Retinal Pigment Epithelial Cells in Macular Degeneration. Ophthalmology.

[bib39] Menon M., Mohammadi S., Davila-Velderrain J., Goods B.A., Cadwell T.D., Xing Y., Stemmer-Rachamimov A., Shalek A.K., Love J.C., Kellis M., Hafler B.P. (2019). Single-cell transcriptomic atlas of the human retina identifies cell types associated with age-related macular degeneration. Nat. Commun..

[bib40] Meyer J.G., Garcia T.Y., Schilling B., Gibson B.W., Lamba D.A. (2019). Proteome and Secretome Dynamics of Human Retinal Pigment Epithelium in Response to Reactive Oxygen Species. Sci. Rep..

[bib41] Miettinen T.P., Björklund M. (2017). Mitochondrial Function and Cell Size: An Allometric Relationship. Trends Cell Biol..

[bib42] Monavarfeshani A., Yan W., Pappas C., Odenigbo K.A., He Z., Segre A.V., van Zyl T., Hageman G.S., Sanes J.R. (2023). Transcriptomic Analysis of the Ocular Posterior Segment Completes a Cell Atlas of the Human Eye. bioRxiv.

[bib43] Moretta A., Bottino C., Vitale M., Pende D., Cantoni C., Mingari M.C., Biassoni R., Moretta L. (2001). Activating receptors and coreceptors involved in human natural killer cell-mediated cytolysis. Annu. Rev. Immunol..

[bib44] Mullin N.K., Voigt A.P., Boese E.A., Liu X., Stone E.M., Tucker B.A., Mullins R.F. (2023). Transcriptomic and Chromatin Accessibility Analysis of the Human Macular and Peripheral Retinal Pigment Epithelium at the Single-Cell Level. Am. J. Pathol..

[bib45] Nagineni C.N., Kommineni V.K., Ganjbaksh N., Nagineni K.K., Hooks J.J., Detrick B. (2015). Inflammatory Cytokines Induce Expression of Chemokines by Human Retinal Cells: Role in Chemokine Receptor Mediated Age-related Macular Degeneration. Aging Dis..

[bib46] Newman A.M., Gallo N.B., Hancox L.S., Miller N.J., Radeke C.M., Maloney M.A., Cooper J.B., Hageman G.S., Anderson D.H., Johnson L.V., Radeke M.J. (2012). Systems-level analysis of age-related macular degeneration reveals global biomarkers and phenotype-specific functional networks. Genome Med..

[bib47] Ortolan D., Sharma R., Volkov A., Maminishkis A., Hotaling N.A., Huryn L.A., Cukras C., Di Marco S., Bisti S., Bharti K. (2022). Single-cell-resolution map of human retinal pigment epithelium helps discover subpopulations with differential disease sensitivity. Proc. Natl. Acad. Sci. USA.

[bib48] Pau G., Fuchs F., Sklyar O., Boutros M., Huber W. (2010). EBImage--an R package for image processing with applications to cellular phenotypes. Bioinformatics.

[bib49] Pombo Antunes A.R., Scheyltjens I., Lodi F., Messiaen J., Antoranz A., Duerinck J., Kancheva D., Martens L., De Vlaminck K., Van Hove H. (2021). Single-cell profiling of myeloid cells in glioblastoma across species and disease stage reveals macrophage competition and specialization. Nat. Neurosci..

[bib50] Postnikova O.A., Rogozin I.B., Samuel W., Nudelman G., Babenko V.N., Poliakov E., Redmond T.M. (2019). Volatile Evolution of Long Non-Coding RNA Repertoire in Retinal Pigment Epithelium: Insights from Comparison of Bovine and Human RNA Expression Profiles. Genes.

[bib51] Qiu T.G. (2019). Transplantation of human embryonic stem cell-derived retinal pigment epithelial cells (MA09-hRPE) in macular degeneration. NPJ Regen. Med..

[bib52] Rabin D.M., Rabin R.L., Blenkinsop T.A., Temple S., Stern J.H. (2013). Chronic oxidative stress upregulates Drusen-related protein expression in adult human RPE stem cell-derived RPE cells: a novel culture model for dry AMD. Aging (Albany NY).

[bib53] Rashid A., Bhatia S.K., Mazzitello K.I., Chrenek M.A., Zhang Q., Boatright J.H., Grossniklaus H.E., Jiang Y., Nickerson J.M. (2016). RPE Cell and Sheet Properties in Normal and Diseased Eyes. Adv. Exp. Med. Biol..

[bib54] Robson J.P., Remke M., Kool M., Julian E., Korshunov A., Pfister S.M., Osborne G.W., Taylor M.D., Wainwright B., Reynolds B.A. (2019). Identification of CD24 as a marker of Patched1 deleted medulloblastoma-initiating neural progenitor cells. PLoS One.

[bib55] Saini J.S., Temple S., Stern J.H. (2016). Human Retinal Pigment Epithelium Stem Cell (RPESC). Adv. Exp. Med. Biol..

[bib56] Salero E., Blenkinsop T.A., Corneo B., Harris A., Rabin D., Stern J.H., Temple S. (2012). Adult human RPE can be activated into a multipotent stem cell that produces mesenchymal derivatives. Cell Stem Cell.

[bib57] Schroder B. (2016). The multifaceted roles of the invariant chain CD74--More than just a chaperone. Biochim. Biophys. Acta.

[bib58] Seel A., Padovani F., Mayer M., Finster A., Bureik D., Thoma F., Osman C., Klecker T., Schmoller K.M. (2023). Regulation with cell size ensures mitochondrial DNA homeostasis during cell growth. Nat. Struct. Mol. Biol..

[bib59] Sharma R., Khristov V., Rising A., Jha B.S., Dejene R., Hotaling N., Li Y., Stoddard J., Stankewicz C., Wan Q. (2019). Clinical-grade stem cell-derived retinal pigment epithelium patch rescues retinal degeneration in rodents and pigs. Sci. Transl. Med..

[bib60] Skeie J.M., Mahajan V.B. (2014). Proteomic landscape of the human choroid-retinal pigment epithelial complex. JAMA Ophthalmol..

[bib61] Song W.K., Park K.M., Kim H.J., Lee J.H., Choi J., Chong S.Y., Shim S.H., Del Priore L.V., Lanza R. (2015). Treatment of macular degeneration using embryonic stem cell-derived retinal pigment epithelium: preliminary results in Asian patients. Stem Cell Rep..

[bib62] Stewart N., Daly J., Drummond-Guy O., Krishnamoorthy V., Stark J.C., Riley N.M., Williams K.C., Bertozzi C.R., Wisnovsky S. (2024). The glycoimmune checkpoint receptor Siglec-7 interacts with T-cell ligands and regulates T-cell activation. J. Biol. Chem..

[bib63] Stoeckius M., Hafemeister C., Stephenson W., Houck-Loomis B., Chattopadhyay P.K., Swerdlow H., Satija R., Smibert P. (2017). Simultaneous epitope and transcriptome measurement in single cells. Nat. Methods.

[bib64] Strunnikova N.V., Maminishkis A., Barb J.J., Wang F., Zhi C., Sergeev Y., Chen W., Edwards A.O., Stambolian D., Abecasis G. (2010). Transcriptome analysis and molecular signature of human retinal pigment epithelium. Hum. Mol. Genet..

[bib65] Sun J., Feng D., Xi H., Luo J., Zhou Z., Liu Q., Chen Y., Shao Q. (2020). CD24 blunts the sensitivity of retinoblastoma to vincristine by modulating autophagy. Mol. Oncol..

[bib66] Takagi S., Mandai M., Gocho K., Hirami Y., Yamamoto M., Fujihara M., Sugita S., Kurimoto Y., Takahashi M. (2019). Evaluation of Transplanted Autologous Induced Pluripotent Stem Cell-Derived Retinal Pigment Epithelium in Exudative Age-Related Macular Degeneration. Ophthalmol. Retina.

[bib67] Thomas B.B., Zhu D., Zhang L., Thomas P.B., Hu Y., Nazari H., Stefanini F., Falabella P., Clegg D.O., Hinton D.R., Humayun M.S. (2016). Survival and Functionality of hESC-Derived Retinal Pigment Epithelium Cells Cultured as a Monolayer on Polymer Substrates Transplanted in RCS Rats. Investig. Ophthalmol. Vis. Sci..

[bib68] Tran N.M., Shekhar K., Whitney I.E., Jacobi A., Benhar I., Hong G., Yan W., Adiconis X., Arnold M.E., Lee J.M. (2019). Single-Cell Profiles of Retinal Ganglion Cells Differing in Resilience to Injury Reveal Neuroprotective Genes. Neuron.

[bib69] Ulicna K., Vallardi G., Charras G., Lowe A.R. (2021). Automated Deep Lineage Tree Analysis Using a Bayesian Single Cell Tracking Approach. Front. Comput. Sci..

[bib70] Voigt A.P., Mulfaul K., Mullin N.K., Flamme-Wiese M.J., Giacalone J.C., Stone E.M., Tucker B.A., Scheetz T.E., Mullins R.F. (2019). Single-cell transcriptomics of the human retinal pigment epithelium and choroid in health and macular degeneration. Proc. Natl. Acad. Sci. USA.

[bib71] Wang Z., Huang X., Lv X., Chen C., Qu S., Ma X., Zhang L., Bi Y. (2022). Bioinformatic analysis identifies potential key genes in the pathogenesis of age-related macular degeneration. Indian J. Ophthalmol..

[bib72] Wei W., Mazzola M., Otero-Marquez O., Tong Y., Souied E., Querques G., Bailey Freund K., Theodore Smith R. (2023). Two potentially distinct pathways to geographic atrophy in age-related macular degeneration characterized by quantitative fundus autofluorescence. Eye (Lond).

[bib73] Whitmore S.S., Wagner A.H., DeLuca A.P., Drack A.V., Stone E.M., Tucker B.A., Zeng S., Braun T.A., Mullins R.F., Scheetz T.E. (2014). Transcriptomic analysis across nasal, temporal, and macular regions of human neural retina and RPE/choroid by RNA-Seq. Exp. Eye Res..

[bib74] Wu S.Z., Al-Eryani G., Roden D.L., Junankar S., Harvey K., Andersson A., Thennavan A., Wang C., Torpy J.R., Bartonicek N. (2021). A single-cell and spatially resolved atlas of human breast cancers. Nat. Genet..

[bib75] Xu Z., Liao X., Li N., Zhou H., Li H., Zhang Q., Hu K., Yang P., Hou S. (2021). A Single-Cell Transcriptome Atlas of the Human Retinal Pigment Epithelium. Front. Cell Dev. Biol..

[bib76] Yan W., Peng Y.R., van Zyl T., Regev A., Shekhar K., Juric D., Sanes J.R. (2020). Cell Atlas of The Human Fovea and Peripheral Retina. Sci. Rep..

[bib77] Yang P., Tyrrell J., Han I., Jaffe G.J. (2009). Expression and modulation of RPE cell membrane complement regulatory proteins. Investig. Ophthalmol. Vis. Sci..

[bib78] Yao J., Jia L., Khan N., Lin C., Mitter S.K., Boulton M.E., Dunaief J.L., Klionsky D.J., Guan J.L., Thompson D.A., Zacks D.N. (2015). Deletion of autophagy inducer RB1CC1 results in degeneration of the retinal pigment epithelium. Autophagy.

[bib79] Zhang S., Wang X.J., Tian L.P., Pan J., Lu G.Q., Zhang Y.J., Ding J.Q., Chen S.D. (2011). CD200-CD200R dysfunction exacerbates microglial activation and dopaminergic neurodegeneration in a rat model of Parkinson's disease. J. Neuroinflammation.

