# Supplementary material for: Single-cell transcriptome and surfaceome profiling of the adult human retinal pigment epithelium
Source: Stem Cell Reports. 2025 Aug 28;20(9):102611. doi: 10.1016/j.stemcr.2025.102611 (PMC12447320; doi:10.1016/j.stemcr.2025.102611)
Supplement: Document S1. Figures S1 and S2 [file mmc1.pdf]

Stem Cell Reports, Volume *20*

Supplemental Information

Single-cell transcriptome and surfaceome profiling of the adult human retinal pigment epithelium

Farhad Farjood, Swapna Nandakumar, Taylor Bertucci, Thomas Kiehl, Steve Lotz, Yue Wang, Jacob Black, Skanda Sai, Jade Kozak, Brigitte L. Arduini, Sally Temple, Nathan C. Boles, and Jeffrey H. Stern

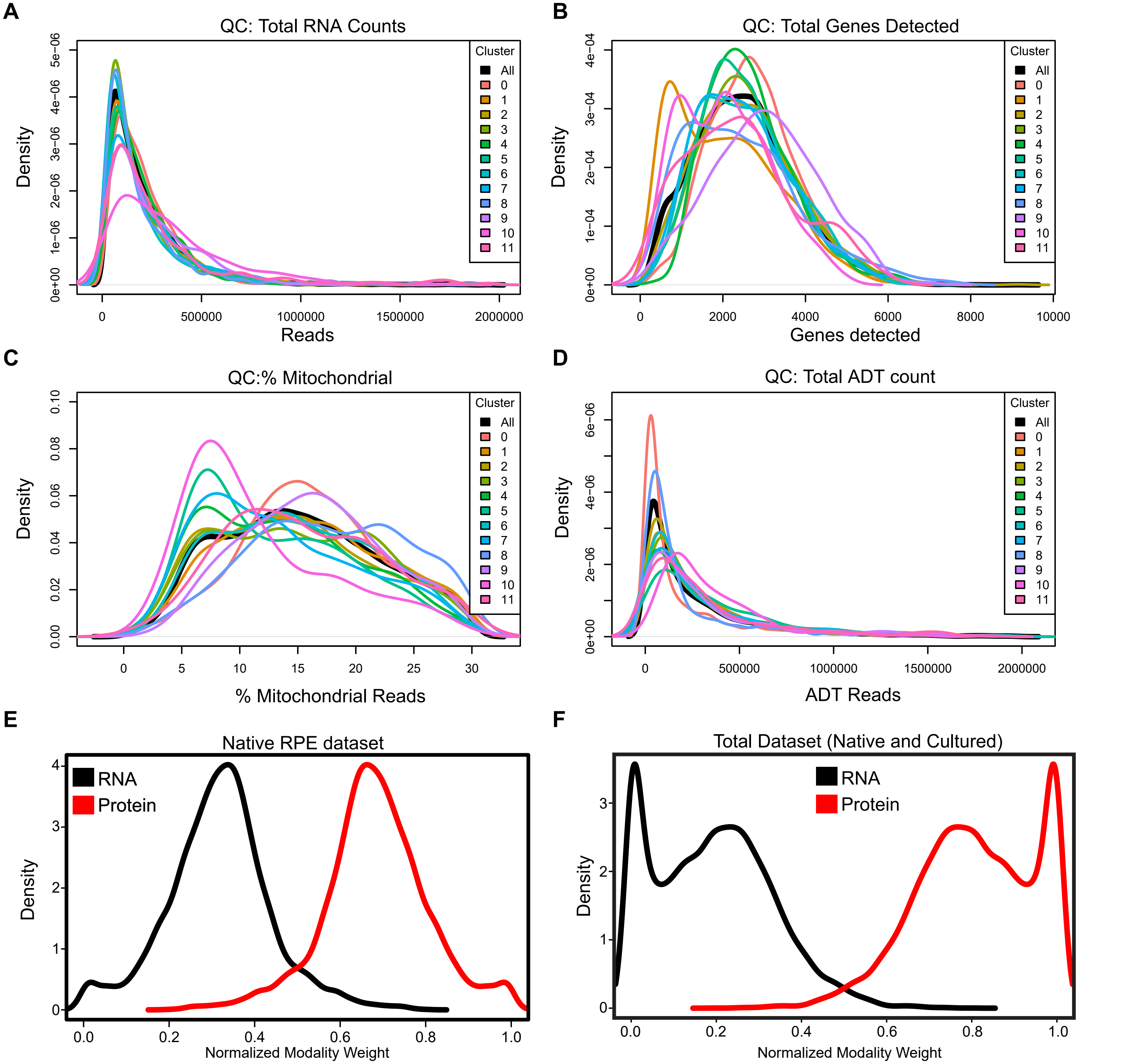

Figure S1. QC measurements and contributions of each modalities to clustering.
We used the well based ICELL8 system for our single cell sequencing. This technology allows for greater read depth per cell and the exclusion of empty wells and wellls with more than one cell. A) Density of reads per cell within each cluster. The miniumun set per cell is 20,000 reads. B) Density of genes detected per cell within each cluster. The minimum set per cell is 200 genes detected C) Density of mitochondrial read percentage per cell within each cluster. Due to the high metabolic activity of the RPE we did not set a filter for this attribute.
D) Density of Antibody derived tag (ADT) reads per cell within each cluster. Normalized modailites weights for each cell in the native RPE (E) and the total dataset (F). Weights are normalized via a softmax transformation to be non-negative and sum to 1.

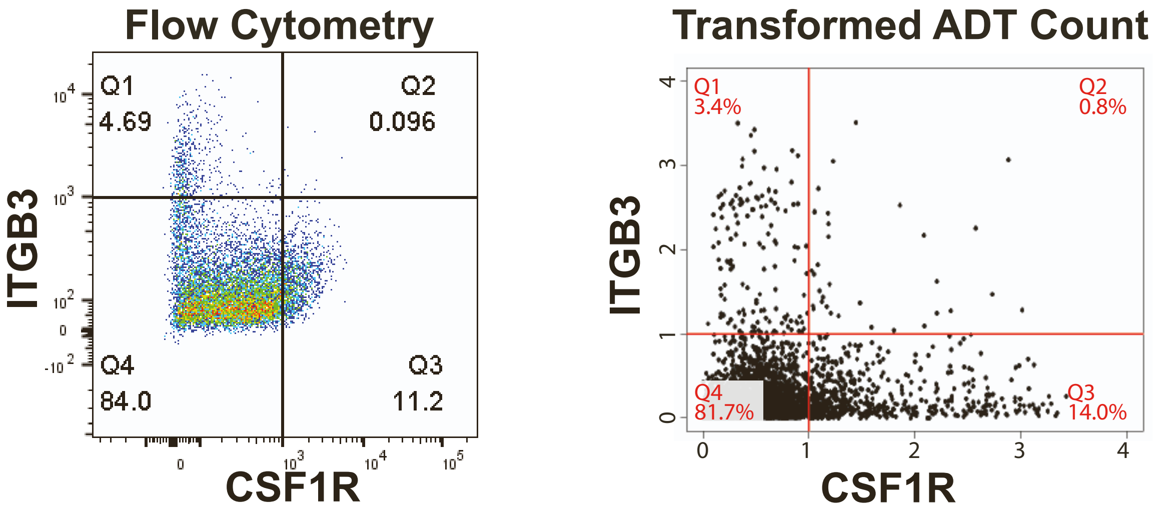

Figure S2. Comparison of FACS results to transformed ADT data.
Similar percentages of cells can be seen in each quadrant.
