# Supplementary material for: Single-cell transcriptome and surfaceome profiling of the adult human retinal pigment epithelium
Source: Stem Cell Reports. 2025 Aug 28;20(9):102611. doi: 10.1016/j.stemcr.2025.102611 (PMC12447320; doi:10.1016/j.stemcr.2025.102611)
Supplement: Document S2. Methods [file mmc2.pdf]

Supplemental Methods

Eye dissection and RPE dissociation

Cultured RPE lines were prepared by culturing dissociated cells for 14-16 weeks followed by cryopreservation using Cryostor CS2 medium (Stem Cell Technologies) at P1. RPE cultures used in CITE-Seq experiments were generated by thawed P1 cells cultured on Transwell inserts for 2, 4 and 10 weeks. RPE cultures were maintained in RPE medium: DMEM F12 50/50 medium (Corning), MEM alpha modification medium (Sigma-Aldrich), 1.25 ml Glutamax (Gibco), 2.5 ml sodium pyruvate (Gibco), 2.5 ml niacinamide (1 M; Spectrum Chemical Inc.), 2.5 ml MEM non-essential amino acid solution (Gibco), 10% heat-inactivated fetal bovine serum (FBS), supplemented with THT (taurine, hydrocortisone, triiodo-thyronin), and 1.25 ml N1 medium supplement (Sigma-Aldrich). Cells were incubated in a humidified incubator at 37°C and 5% CO2. RPE medium with 10% FBS was used until the cultures were confluent and 2% FBS was used after confluency. The medium was replaced every 3 days. Cultures were tested for mycoplasma every 6 weeks PCR and examined regularly for contaminants.

Cryopreservation and thawing

RPE cells were pelleted in a cryovial, supernatant removed, 1-2 mls of cold Cryostor CS2 freezing media added and triturate to break up the pellet, then additional cold CS2 was added to get the desired concentration of cells (e.g.,$1x10^6$cells/ml). Cryovials were transferred to a pre-cooled (4C) Freezer Buddy and placed into a -80 freezer overnight, then transferred to liquid nitrogen. Cells were thawed in 37C water bath, washed with fresh TAB2 media with 10% FBS and plated for culturing.

CITE-Seq library preparation

To prepare ADT libraries, an ADT additive primer (0.1 ng) was introduced into the RT-PCR reaction. Following PCR product extraction, cDNA samples underwent concentration and purification steps using a DNA Clean & Concentrator-5 kit (Zymo Research, Irvine, CA) and

were further purified with a 0.6X proportion of AMPure XP magnetic beads (Beckman Coulter, Brea, CA) following manufacturer protocols. Subsequently, ADT libraries were constructed from the supernatant of the initial AMPure XP purification utilizing 2X AMPure XP magnetic beads. Amplification and purification of the purified cDNA samples were carried out using a Nextera XT DNA Library Preparation Kit (Illumina, San Diego, CA) in accordance with the instructions provided in Takara's 3' DE chip and reagent kit (Biolegend). ADT libraries underwent amplification using RPI-X primers containing the P7 sequence and were then purified using 2X AMPure XP beads. The concentration of cDNA products was determined using a Qubit Fluorometer with the Qubit dsDNA HS Assay Kit (Thermo Fisher Scientific, Waltham, MA). Quality assessment of the cDNA and ADT product was conducted using an Agilent High Sensitivity DNA Kit and Agilent 2100 Bioanalyzer (Agilent Technologies, Palo Alto, CA) to ensure the complete removal of contaminants. Libraries were sequenced on NovaSeq 6000 high-output flow cell, generating 2 × 150-bp read lengths (GeneWiz).

Data processing and analysis

Raw Illumina read BCL Files were converted to fastq files using the bcl2fastq2 software (bcl2Fastq v2.19.1, Illumina, Inc). Fastq files were then merged into read1 (ICELL8 barcode sequence) and read2 (transcript sequence) fastq files. Read were mapped to human hg38 genome using the Cogent NGS Analysis Pipeline (V1.5, Takara Bio). The resulting transcript and ADT read count matrices were used as the input for further analysis with Seurat (V4 and V5) package for R. The counts matrix was converted to a Seurat object using CreateSeuratObject function with a minimum cell cutoff of 3 and a minimum feature cutoff of 200.
